# Expectations, Emotions, and Empowerment: Understanding Nurses’ Needs in the Age of AI

**DOI:** 10.1016/j.mnl.2025.102490

**Published:** 2025-09-22

**Authors:** Jiyoun Song

**Affiliations:** Department of Biobehavioral Health Sciences, University of Pennsylvania School of Nursing, Philadelphia, Pennsylvania.

The integration of artificial intelligence (AI) into health care is not a future concept; it is already reshaping workflows through decision support, documentation tools, and virtual assistants.^1,2^ Yet, technological readiness does not ensure human readiness. Nurses show varied reactions to AI, from enthusiasm to resistance.^3,4^ However, the emotional dynamics surrounding AI adoption are not entirely new, recalling past transitions like the electronic medical record systems rollout. Then, as now, concerns focused more on disruption than utility of electronic medical records.^5^ Importantly, such reactions should not be interpreted as indicators of unwillingness or lack of competence. Rather, they represent natural human responses to organizational change, particularly when that change is perceived as threatening safety, autonomy, or professional identity. To address these challenges, nurse leaders need psychological insight as well as technical plans. This paper offers a framework combining Gartner’s Hype Cycle with Maslow’s Hierarchy of Needs to guide emotionally informed leadership in AI integration.

## Two Models to Understand Emotional Adoption

**Figure 1.**
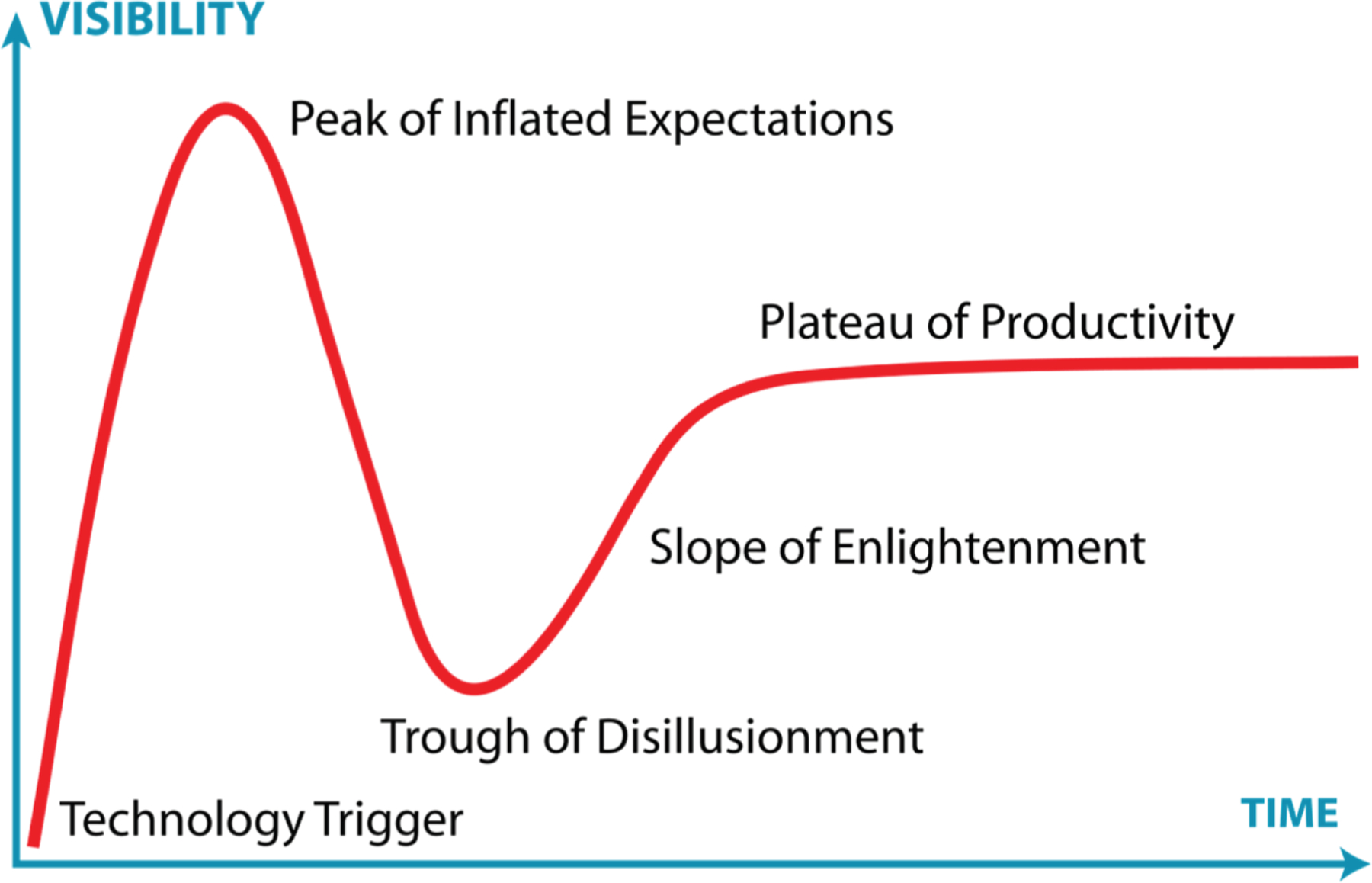
Gartner’s Hyper Cycle. Adapted from Jeremykemp, 2007, Wikimedia Commons, CC BY-SA 3.0^6^

(1) Gartner’s Hype Cycle (See Figure 1)^7^ : this model describes a common pattern of technology adoption—early excitement (innovation trigger), rising but often unrealistic enthusiasm (peak of inflated expectations), disillusionment as challenges emerge (trough of disillusionment), followed by gradual learning (slope of enlightenment), and finally, sustained, productive use (plateau of productivity).

(2) Maslow’s Hierarchy of Needs: this model helps explain why people respond as they do. Maslow describes five levels of human needs: physiological, safety, belonging, esteem, and self-actualization.^8^ New technologies can either support or threaten these needs.

## The Emotional Landscape of AI Adoption in Nursing and Strategies for Nurse Leaders

The stages below align Gartner’s timeline with the most salient human needs at each phase, showing how these frameworks can guide leadership attention.

(1) Initial awareness and curiosity (innovation trigger → minimal or latent need activation): AI interest begins with broad exposure through leadership messages, organizational initiatives, or media coverage before it becomes personally relevant. Engagement is abstract, and psychological needs remain mostly latent.

- Leadership strategy: spark interest with storytelling and informal exposure. Focus on exploration, not commitment.

(2) Hope and anticipation (peak of inflated expectations → physiological + belonging + esteem needs): at the peak of inflated expectations, organizational and industry enthusiasm for AI is high. At the peak of inflated expectations, nurses may feel optimistic about AI, especially when early successes are highlighted and the promise of efficiency and professional recognition is emphasized. These reactions can activate multiple levels of need: physiological (e.g., hope for reduced workload), safety (e.g., desire for job clarity), belonging (e.g., inclusion in innovation efforts), and esteem (e.g., recognition for embracing change). However, temporary enthusiasm rooted in esteem and a sense of belonging may fade if basic needs, such as safety or rest, go unmet. At this stage, excitement is fragile, built more on inflated hopes than real support.

- Leadership strategy: manage expectations while validating hope. Emphasize realistic outcomes and share lessons from early adopters. Acknowledge both excitement and anxiety and connect AI initiatives to existing nursing values.

(3) Frustration and withdrawal (trough of disillusionment → physiological + safety + esteem needs): When AI creates inefficiencies or errors, nurses may lose confidence in the system and themselves. In some cases, technical glitches can cause physical fatigue, extend shifts, and affect basic physiological needs such as rest and recovery. Disengagement may result from frustration, particularly if their professional judgment feels sidelined.

- Leadership strategy: normalize frustration as part of the learning process. Offer hands-on support and space to give feedback without judgment. Clarify that nurses’ expertise remains essential and build confidence with quick wins.

(4) Recovery and rebuilding (slope of enlightenment → reclaiming esteem + belonging needs): through constructive feedback, peer support, and iterative training, nurses begin rebuilding trust in the system. When they feel heard and valued in this learning phase, both esteem and team connection are restored.

- Leadership strategy: involve nurses in workflow redesign and celebrate progress. Reinforce shared ownership and peer mentorship.

(5) Integration and empowerment (plateau of productivity → esteem + self-actualization needs): as AI becomes part of routine practice, empowered nurses move beyond basic use toward innovation and leadership. They integrate the tools in ways that express their clinical judgment, mentor others, and contribute meaningfully to patient care, meeting higher-level needs for esteem and fulfillment.

- Leadership strategy: sustain engagement by aligning AI with purpose. Recognize nurse-led innovation and support professional growth.

## CONCLUSION:HUMANIZING THE AI JOURNEY

AI adoption is not merely about implementing new tools. It requires understanding and guiding the human experience of change. Like past shifts to EHRs, success hinges on how well organizations support users. From this paper, nursing leaders can anticipate resistance, build trust, and deal with emotional realities based on Gartner’s Hype Cycle and Maslow’s Hierarchy of Needs during implementation. This approach complements emerging frameworks such as the Innovativeness Across Academia and Practice for Healthcare Progress Scale,^9^ which emphasize relational and structural foundations of successful innovation. Leading with emotional fluency and strategic empathy ensures nurses do not just adapt to change—they thrive in it, shape it, and improve care through it. The future of AI in nursing will be defined not by speed of adoption, but by the strength of human-centered support.
